# Epigenetic regulation of thyroid hormone-induced adult intestinal stem cell development during anuran metamorphosis

**DOI:** 10.1186/2045-3701-4-73

**Published:** 2014-11-28

**Authors:** Guihong Sun, Liezhen Fu, Yun-Bo Shi

**Affiliations:** School of Basic Medical Sciences, Wuhan University, Wuhan, 430072 P.R. China; Section on Molecular Morphogenesis, Program in Cellular Regulation and Metabolism (PCRM), Eunice Kennedy Shriver National Institute of Child Health and Human Development (NICHD), National Institutes of Health (NIH), 18 Library Dr, Bethesda, Maryland 20892 USA

**Keywords:** Thyroid hormone receptor, Stem cell, Metamorphosis, *Xenopus laevis* and *tropicalis*, Histone methylation, Histone acetylation, Intestine

## Abstract

Epigenetic modifications of histones are emerging as key factors in gene regulation by diverse transcription factors. Their roles during vertebrate development and pathogenesis are less clear. The causative effect of thyroid hormone (T3) on amphibian metamorphosis and the ability to manipulate this process for molecular and genetic studies have led to the demonstration that T3 receptor (TR) is necessary and sufficient for *Xenopus* metamorphosis, a process that resembles the postembryonic development (around birth) in mammals. Importantly, analyses during metamorphosis have provided some of the first in vivo evidence for the involvement of histone modifications in gene regulation by TR during vertebrate development. Furthermore, expression and functional studies suggest that various histone modifying epigenetic enzymes likely participate in multiple steps during the formation of adult intestinal stem cells during metamorphosis. The similarity between intestinal remodeling and the maturation of the mammalian intestine around birth when T3 levels are high suggests conserved roles for the epigenetic enzymes in mammalian adult intestinal stem cell development and/or proliferation.

**Electronic supplementary material:**

The online version of this article (doi:10.1186/2045-3701-4-73) contains supplementary material, which is available to authorized users.

## Introduction

The adult mammalian intestine has long been served as a model system to study the property and function of adult organ-specific stem cells due to the constant self-renewal of the intestinal epithelium throughout adult life[1, 2]. In the adult mammalian intestine, the stem cells reside in the crypts. After stem cell division, the daughter cells migrate along the crypt-villus axis as they gradually differentiate into different types of epithelial cells. At the tip of the villus, the differentiated epithelial cells undergo apoptosis and are replaced by the newly arrived, differentiated epithelial cells, completing the self-renewing cycle once every 1–6 days[2–4]. Similar processes occur in the intestine in all vertebrates, including amphibians, with the epithelium being replaced once every 2 weeks in *Xenopus laevis*[5]. While many studies have been carried out on the mammalian intestinal stem cells in the adult, few have been on the formation of such stem cells during vertebrate development, largely due to the difficulty to manipulate uterus-enclosed mammalian embryos.

**Figure 1:**
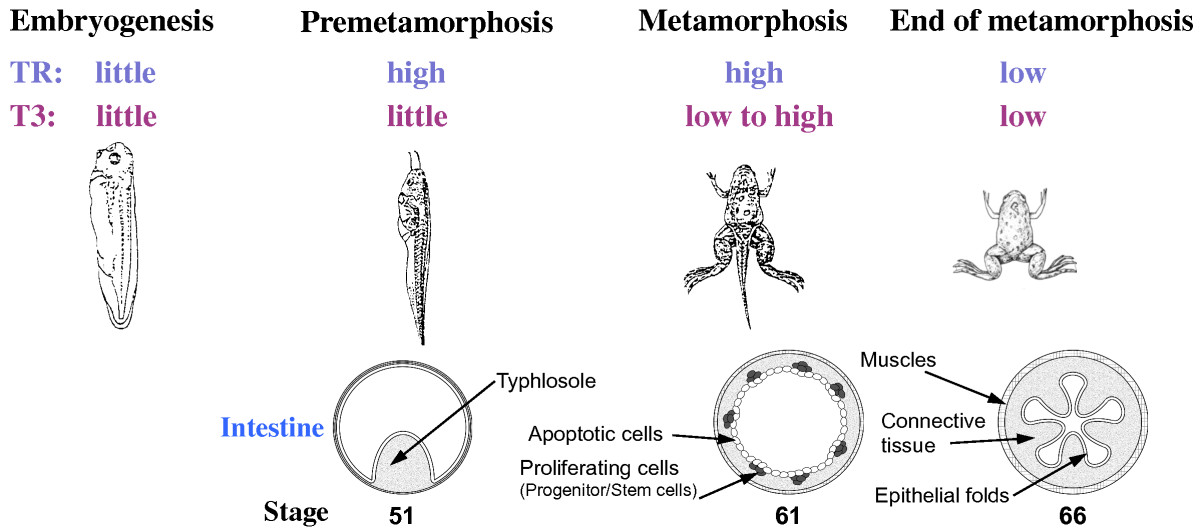
**T3-dependent intestinal remodeling during** ***Xenopus*** **metamorphosis involves larval cell apoptosis and de novo formation of adult epithelial stem cells.** *Xenopus* undergoes a biphasic development. Its embryogenesis, when there is little TR or T3, leads to the formation of a free-living premetamorphic tadpoles by stage 45. During premetamorphosis (stage 45–54), there are high levels of TR but little T3, and the intestine has a simple structure with only a single fold, the typhlosole. During metamorphosis, the T3 level in the plasma rises to peak around stage 62, and most larval epithelial cells in the intestine undergo apoptosis, as indicated by the circles. Concurrently, the proliferating adult progenitor/stem cells are formed *de novo* from larval epithelial cells through dedifferentiation, as indicated by black dots. By the end of metamorphosis (stage 66), the levels of both TR and T3 drop lower and the newly differentiated adult epithelial cells in the intestine form a multiply folded epithelium.

The frog intestine resembles the adult intestine in mammals. In the highly related species *Xenopus laevis and tropicalis*, the frog intestine contains numerous epithelial folds that resemble the crypt-villus structure in mammals[6, 7]. The stem cells localized in the trough of the fold proliferate and the daughter cells differentiate into different epithelial cells as they migrate up toward the crest of the fold, where they undergo apoptosis. Interestingly, amphibians undergo biphasic development, first forming a free-living tadpole (Figure 1). After a finite period of premetamorphic growth, the tadpole metamorphoses into a frog. Accompanying this metamorphic transition, the animal intestine remodels extensively. The *Xenopus* tadpole intestine is a simple tubular structure made of mainly larval epithelial cells with little connective tissue or muscles (Figure 1). It has a single epithelial fold, the typhlosole. During metamorphosis, the vast majority of the epithelial cells undergo apoptosis while some differentiated larval epithelial cells dedifferentiate into adult progenitor/stem cells, which subsequently proliferate and differentiate to form a multi-folded adult epithelium surrounded by extensive connective tissue and muscles[1, 8–12]. As metamorphosis occurs totally independently of maternal influence, this offers a unique opportunity to study how adult organ-specific stem cells are formed during vertebrate development.

## Thyroid hormone (T3) and the formation of adult intestinal stem cells

Both the maturation of the adult mammalian intestine and the remodeling of the intestine during frog metamorphosis occur when the plasma thyroid hormone (T3) concentrations are high, a period referred to as postembryonic development[13]. Importantly, T3 plays a causative and organ-autonomous role during amphibian metamorphosis[14, 15]. T3-treatment of premetamorphic tadpoles or tadpole organ cultures induces precocious metamorphosis while blocking the synthesis of endogenous T3 inhibits natural metamorphosis. This has enabled cellular, molecular, and genetic analyses on the formation of the adult intestinal stem cells during intestinal metamorphosis[1, 16–18]. By using recombinant organ-cultures made of wild type and transgenic animals expressing GPF, we have shown that adult epithelial stem cells formed upon T3 treatment of the organ cultures of premetamorphic intestine originate from the larval epithelium[8].

Extensive studies indicate that T3 controls *Xenopus* metamorphosis by regulating gene transcription through nuclear T3 receptors (TRs)[19–31]. To investigate the role of TR in adult intestinal stem cell development, we have made use of recombinant organ-cultures consisting of tissues from wild type and transgenic animals expressing a dominant positive TR (dpTR) under the control of a heat shock-inducible promoter[10, 22]. We have shown that inducible expression of the dpTR in all tissues of the intestine in the absence of T3 is sufficient to induce intestinal metamorphosis, including larval epithelial cell death and adult stem cell formation, suggesting that TR is both necessary and sufficient for the inductive effects of T3 on stem cell formation[10]. Furthermore, expression of dpTR in the larval epithelium alone is able to induce the dedifferentiation of larval epithelial cells to upregulate sonic hedgehog gene, which is highly expressed in the proliferating adult epithelial progenitor/stem cells. Interestingly, such cells fail to upregulate the expression of two well-known markers of the adult mammalian intestinal stem cells and the formation of the stem cells expressing such markers also requires the expression of dpTR in the rest of the intestinal organ culture, i.e., the non-epithelium[10], consistent with earlier studies showing an requirement for cell-cell interaction during the formation of the adult intestine[28, 32]. These findings suggest that TR-mediated gene regulation in both the epithelium and the non-epithelium are required for stem cell development, with the T3-induced gene expression changes in the non-epithelium likely contribute to the formation of the stem cell niche for the developing adult stem cells. Many such tissue-specific T3-regulated genes have been identified and the analyses of the spatiotemporal expression profiles of some of the epithelial genes indeed support their involvement in adult stem cell formation/proliferation[33–37].

## Mechanism of gene regulation by TR during *Xenopus* development

TR can both activate and repress gene transcription. For T3-induced genes, TR most likely functions as heterodimers formed with 9-cis retinoic acid receptors (RXRs), another number of the nuclear hormone receptor superfamily[38–42]. TR/RXR heterodimers bind to T3-response elements (TREs) in target genes constitutively and regulates their expression in a T3-dependent manner[38–41, 43–45]. In the absence of T3, TR binds to corepressors such as the two highly related proteins N-CoR (nuclear corepressor) and SMRT (silencing mediator of retinoid and thyroid hormone receptors), which form large histone deacetylase (HDAC)-containing complexes[46–62]. In the presence of T3, TR recruits diverse coactivator complexes, such as ATP-dependent chromatin remodelers and histone acetyltransferase/methyltransferase-containing complexes[39, 51, 63–84]. Thus, TR likely regulates gene transcription in part through chromatin remodeling and histone modifications.

**Figure 2:**
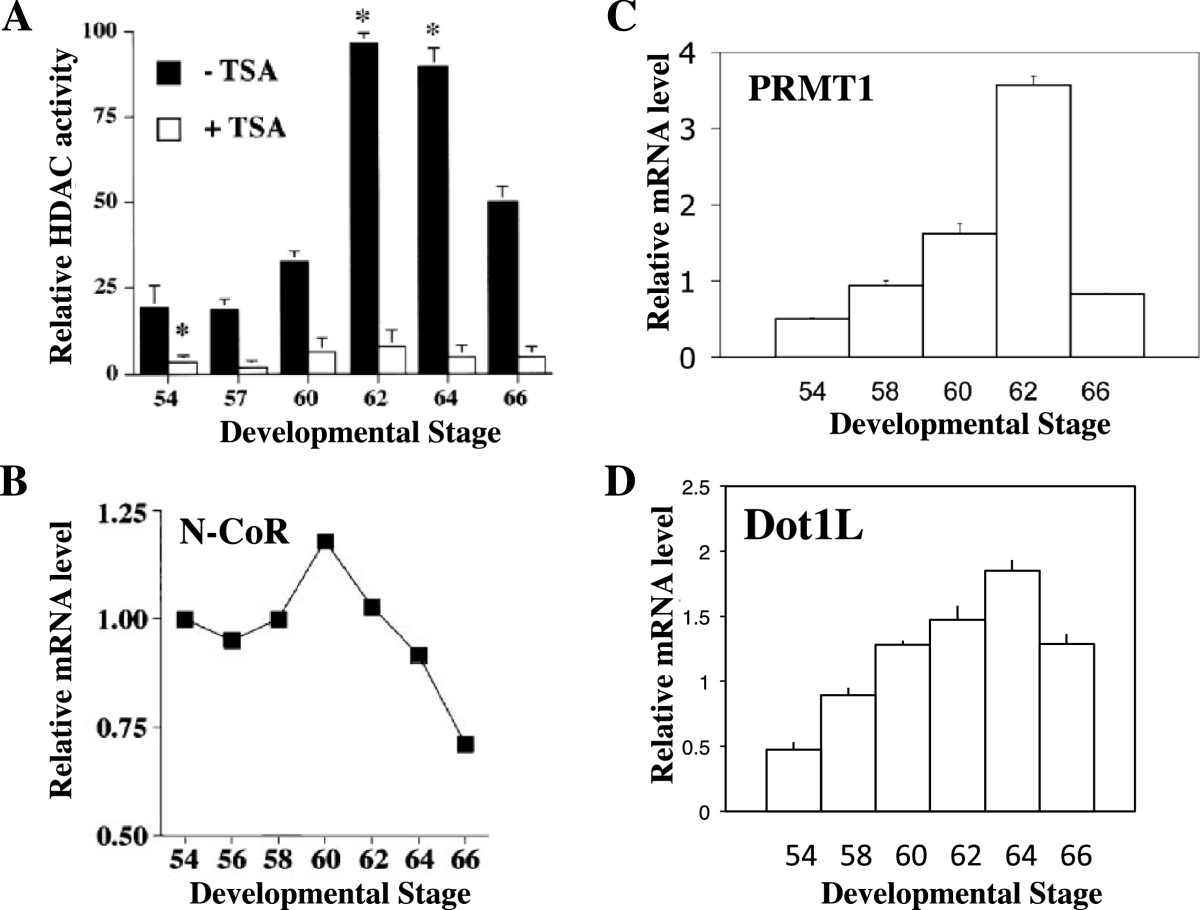
**Upregulation of genes involved in epigenetic modifications during intestinal stem cell development. (A)** HDAC activity. Intestinal protein extracts were prepared from *Xenopus laevis* tadpoles at different stages and assayed for HDAC activity in the presence or absence of 10 nM TSA, an HDAC inhibitor. Means +/- SEMs are given. Statistical significance as compared with the stage 54 animals is expressed as *: P <0.01. Note that the HDAC-specific drug TSA inhibited all activities. See[91] for details. **(B)**-**(D)**. The relative mRNA levels of N-CoR **(B)**, PRMT1 **(C)**, and Dot1L **(D)**. The mRNA levels were determined by using total RNA from intestine at different stages during *Xenopus laevis* development. See[76, 87, 98] for details.

Molecular studies during frog development were the first to provide strong evidence for the involvement of epigenetic changes in gene regulation by TR during vertebrate development. First, chromatin immunoprecipitation (ChIP) assay has shown that TR and RXR bind to T3-inducible genes constitutively in pre- and metamorphosing *Xenopus laevis* and *tropicalis* tadpoles[85, 86]. Second, gene regulation by T3 during T3-induced as well as natural metamorphosis is accompanied by increases in the histone acetylation levels at the target genes as well as the release of corepressor complexes and the recruitment of coactivator complexes[62, 76, 81–85, 87–89]. More importantly, treatment premetamorphic tadpoles with the HDAC inhibitor tricostatin A (TSA) inhibits HDAC activity in tadpole tissues and derepresses T3-response genes in the absence of T3 (Figures 2 and3)[90, 91]. Finally, ChIP analyses of total histones and different histone acetylation and methylation marks have shown that T3 treatment leads to the removal of core histones at the T3 target genes, and a reduction in the levels of repression histone modification marks and an increase in the levels of activation histone modification marks in the remaining nucleosomes[31, 88, 89, 92]. This is consistent with earlier studies in the reconstituted frog oocyte transcription system, where the ordered nucleosomal organization of the minichromosome containing a T3-responsive promoter assembled in the *Xenopus laevis* oocyte, was found to be disrupted by TR/RXR in the presence but not in the absence of T3[43, 44, 93, 94]. The exact mechanisms for the chromatin remodeling are yet to be determined. It is likely that the release of the HDAC-containing N-CoR/SMRT complexes contributes to the increased acetylation at the target genes. Likewise, the recruitment of the coactivator complexes, such as the chromatin remodeling complexes containing Brg1 and BAF57 and histone modification complexes containing acetyltransferases SRC and p300 and methyltransferases PRMT1 and CARM1, would help to remodel the chromatin and alter histone modifications at the promoter regions[21, 31, 62–64, 76, 77, 81–85, 87–89].

**Figure 3:**
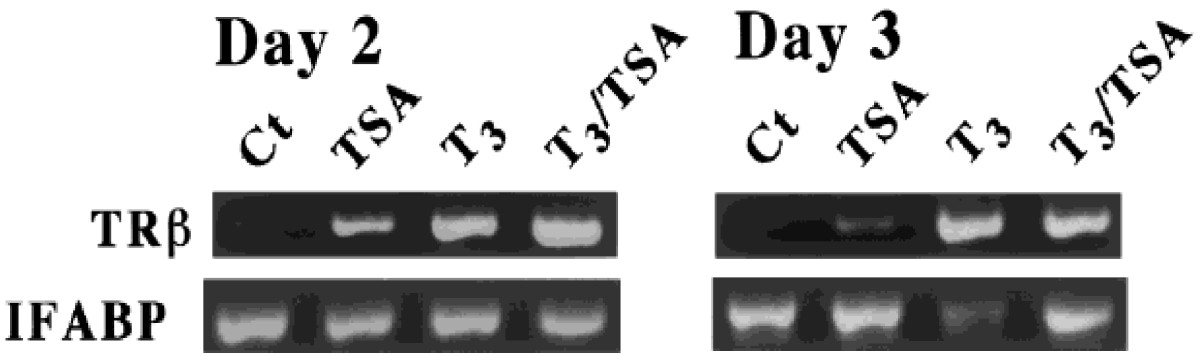
**TSA induces direct TR target genes but blocks the regulation of late T**_**3**_**-response genes in premetamorphic tadpole intestine.** Stage 55 tadpoles were treated with T_3_ (5 nM) and/or TSA (100 nM) for the indicated number of days. Total RNA was extracted from isolated intestine and assayed by PCR for the mRNA levels of indicated genes. IFAPB: intestinal fatty acid binding protein. See[91] for details.

## Regulation of genes encoding epigenetic enzymes during intestinal stem cell development

The changes in the levels of various histone modifications upon gene activation by T3 argue for a role of epigenetic genes during adult intestinal stem cell development. Interestingly, when HDAC activity and HDAC1 (Rpd3) expression were analyzed in the metamorphosing intestine, both were found to be low in premetamorphic tadpoles (Figure 2A)[91] and strongly upregulated at the climax of metamorphosis (stages 60–62) when stem cells are forming and proliferating[91]. Similar observation was made for the expression of the TR-binding corepressor N-CoR (Figure 2B)[87], which forms complexes with HDACs[56, 61, 95]. Thus, it is likely that in addition to their roles in facilitating repression by unliganded TR (see above), the HDAC-containing corepressor complexes may also play a role during metamorphosis when T3 is present (see below). Among the histone acetyltransferases analyzed, SRC3 were found to be upregulated during intestinal metamorphosis while SRC1 and p300 changed little during metamorphosis in the intestine[96].

In addition, several histone methyltransferases are also expressed in the metamorphosing intestine. The histone H3R17 methyltransferase CAMR1 is expressed constitutively during metamorphosis[77] while the histone H4R3 methyltransferase PRMT1 is upregulated by T3 during both natural and T3-induced intestinal metamorphosis (Figure 2C)[76]. More recent promoter analyses have suggested that PRMT1 is indirectly induced by T3, in part through the activation of c-Myc gene[97], a transcription factor that is known to be important for stem cells and cell proliferation in general. Another histone methyltransferase, Dot1L (Dot1-like), the only known histone H3K79 methyltransferase**,** has been shown to be upregulated in the intestine during metamorphosis (Figure 2D) and its induction is directly at the transcription level through the binding of TR to a TRE in its promoter[98]. Thus, multiple histone methyltransferases appear to be involved in the adult intestinal stem cell development.

## Distinct roles of epigenetic enzymes at multiple steps of intestinal stem cell development

As indicated above, unliganded TR recruits HDAC-containing corepressor complexes to T3-target genes in different organs of premetamorphic tadpoles, including the intestine, while liganded TR recruits coactivator complexes containing histone acetyltransferases and methyltransferases. These enable TR to play a dual function role during frog development, repressing T3-inducible genes to prevent premature metamorphosis in the absence of T3 while activating these genes to induce metamorphosis when T3 is present[24]. The involvement of HDAC(s) in gene repression by unliganded TR has been substantiated by the ChIP analyses on histone acetylation levels at the T3 target genes[90, 91]. Furthermore, overexpression of a dominant negative corepressor N-CoR that disrupts the formation of an active HDAC-containing corepressor complex at T3 target genes results in precocious initiation of metamorphosis and the upregulation of T3 target genes[99]. Thus, HDAC activity plays an important role to repress TR target genes in the premetamorphic tadpole intestine to prevent precocious formation of adult stem cells. Interestingly, the expression of N-CoR and HDAC1 as well as HDAC activity is strongly upregulated during intestinal metamorphosis (Figure 2). Thus, HDAC activity is likely also important for one or more steps downstream of gene activation by liganded TR. This dual role of HDACs in intestinal development has been supported by molecular studies using the HDAC inhibitor TSA. TSA treatment of premetamorphic tadpoles for 2–3 days in the absence of T3 leads to upregulation of T3-target genes such as TRβ, while in the presence of T3, little effect is observed (Figure 3)[91]. This agrees with the mechanism that unliganded TR represses TR target genes by recruiting HDAC-containing complexes and inhibiting HDACs will thus depress the genes. In the presence of T3, the HDAC-complexes are released from the T3 target promoters and thus inhibiting HDAC will have no effect. Interestingly, TSA surprisingly inhibits T3-induced metamorphosis and the regulation of down-stream T3 response genes[91]. For example, in the animal intestine, the formation and/or proliferation of the adult epithelial stem cells is inhibited by TSA treatment[91]. Likewise, the downregulation of intestinal fatty acid binding protein (IFABP) gene after prolonged T3-treatment, which accompanies larval epithelial cell death and adult stem cell development, is also blocked by TSA (Figure 3). Thus, histone deacetylation appears to also function at a step(s) down-stream of gene regulation induced by liganded TR to affect the regulation of genes involved in the subsequent steps important for adult intestinal stem cell formation.

Among the histone methyltransferases known to be expressed during intestinal metamorphosis, both CARM1 and PRMT1 are TR-coactivators and likely act at least in part to enhance the transcriptional regulation by liganded TR to promote adult stem cell development. Indeed, transgenic overexpression of wild type PRMT1 leads to an increased number of intestinal stem cells during metamorphosis while antisense morpholino-mediated PRMT1 knockdown reduces the number of such stem cells[11]. Thus, PRMT1 is important for the formation and/or proliferation of adult intestinal progenitor/stem cells during metamorphosis. Mechanistically, we have shown that overexpression of PRMT1 indeed enhances the activation of T3-target genes in the presence of T3 in tadpoles. On the other hand, it is very likely that PRMT1 can also function to epigenetically influence the expression of genes regulated by some other transcription factors during stem cell development.

The third methyltransferase, Dot1L, is the only known histone methyltransferase capable of methylating histone H3K79[100]. Interestingly, ChIP analyses have revealed that the levels of H3K79 methylation at T3 target promoters are strongly increased during either natural or T3-induced metamorphosis in the intestine[89]. These findings suggest that T3 activates the Dot1L gene, and Dot1L in turn feeds back positively as a TR coactivator during metamorphosis by methylating H3K79 at T3 target genes to enhance gene activation and intestinal stem cell development. On the other hand, like PRMT1, Dot1L may also influence the activity of other transcription factors during intestinal metamorphosis.

## Conclusion

Ever increasing evidence supports the view that histone modifications are key epigenetic marks that can influence gene expression during development and pathogenesis. Each eukaryotic nucleosome contains four core histones (H2A, H2B, H3, and H4). These histones, particularly their N-terminal tails, are subject to various posttranslational modifications, including acetylation and methylation, etc.[101]. A number of histone activation and repression marks have been identified based on the correlations of histone modifications at individual genes with the levels of the corresponding mRNAs as determined by genome wide ChIP and gene expression analyses in cell cultures[102–110]. The total dependence of amphibian metamorphosis on T3 and TR and the ability to easily manipulate this process for molecular and genetic studies[20, 23, 31, 111] have enabled the analyses of some of these modifications in vivo. These studies have shown that most histone modification marks, although not all, are similarly correlated with gene regulation by TR during *Xenopus* metamorphosis and adult intestinal stem cell development[88, 89, 92], suggesting that TR utilizes such epigenetic modifications to control gene expression during vertebrate development. Importantly, the distinct spatiotemporal expression profiles of various epigenetic enzymes during intestinal remodeling implicates complex roles of epigenetic enzymes during adult intestinal stem cell development. In particularly, HDAC activity appears to be required not only by unliganded TR to prevent precocious intestinal metamorphosis in premetamorphic tadpoles but also at one or more steps downstream of gene activation by liganded TR for adult intestinal stem cell development. Similarly, the histone methyltransferases CARM1, PRMT1, and Dot1L are likely involved both as coactivators for TR and in the downstream events leading to the formation of adult intestinal stem cells. Interestingly, a number of studies have also revealed the importance of epigenetic modifications for other adult organ-specific stem cells[112–114]. Clearly, functional studies by using overexpression and knockdown approaches in vivo[115–119] are needed to determine the exact roles of these epigenetic enzymes for *Xenopus* intestinal stem cell development. Furthermore, the similarity between amphibian metamorphosis and postembryonic development (the period around birth when T3 levels are high)[13, 15], and in particular between intestinal metamorphosis and mammalian intestinal maturation[17, 120], suggests conserved roles for the epigenetic enzymes in the formation and/or proliferation of adult vertebrate intestinal stem cells.

## Authors’ original submitted files for images

Below are the links to the authors’ original submitted files for images. Authors’ original file for figure 1 Authors’ original file for figure 2 Authors’ original file for figure 3
