# Supplementary material for: Epigenetic regulation of thyroid hormone-induced adult intestinal stem cell development during anuran metamorphosis
Source: Cell Biosci. 2014 Nov 28;4:73. doi: 10.1186/2045-3701-4-73 (PMC4417507; doi:10.1186/2045-3701-4-73)
Supplement: Supplementary file 1 — Authors’ original file for figure 1 [file 13578_2014_210_MOESM1_ESM.pdf]

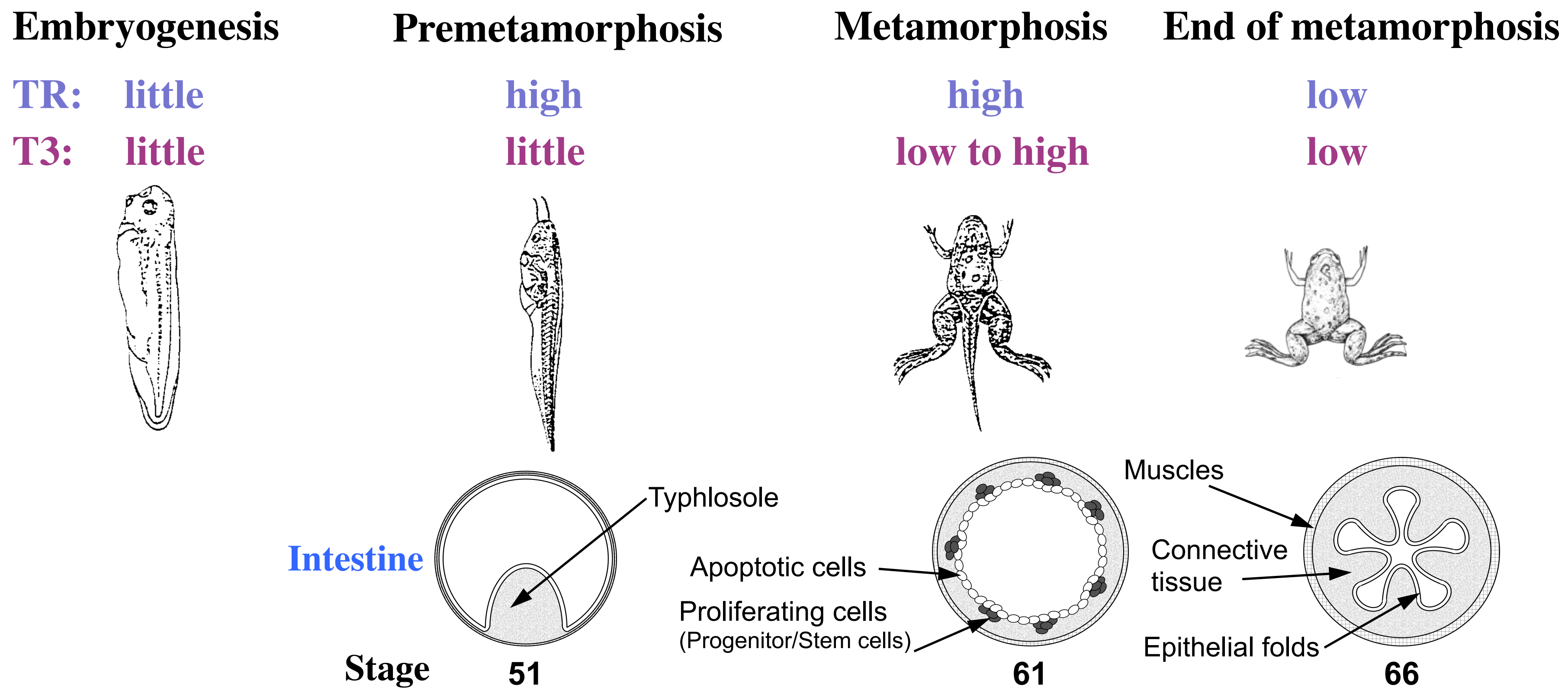
Embryogenesis
Premetamorphosis
Metamorphosis
End of metamorphosis
TR:
little
high
high
low
T3:
little
little
low to high
low
Intestine
Typhlosole
Apoptotic cells
Proliferating cells
(Progenitor/Stem cells)
Muscles
Connective tissue
Epithelial folds
Stage
51
61
66
