# Supplementary figures and images for: Epigenetic regulation of thyroid hormone-induced adult intestinal stem cell development during anuran metamorphosis
Source: Cell Biosci. 2014 Nov 28;4:73. doi: 10.1186/2045-3701-4-73 (PMC4417507; doi:10.1186/2045-3701-4-73)

**A**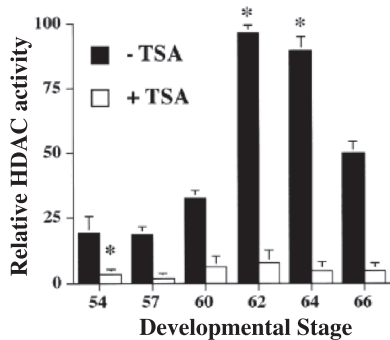**B**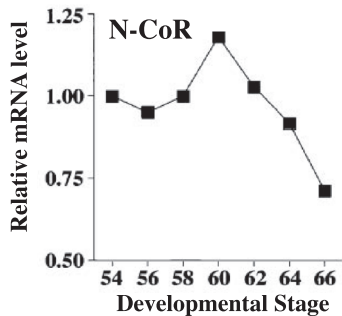**C**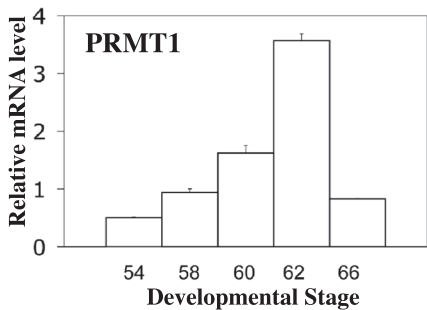**D**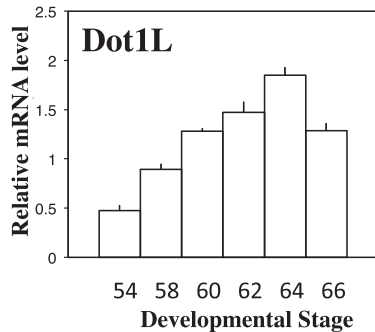

Supplement: Supplementary file 2 — Authors’ original file for figure 2 [file 13578_2014_210_MOESM2_ESM.pdf]

**Day 2**

**Ct**

**TSA**

**T3**

**T3/TSA**

**Day 3**

**Ct**

**TSA**

**T3**

**T3/TSA**

**TR $\beta$**

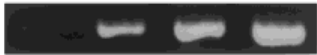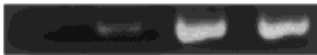

**IFABP**

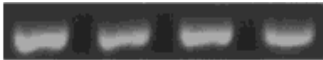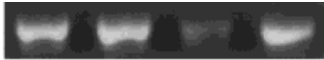

Supplement: Supplementary file 3 — Authors’ original file for figure 3 [file 13578_2014_210_MOESM3_ESM.pdf]
